# Medium-term consequences (5 years) of the Covid-19 crisis on work organization and occupational risks: a prospective study

**DOI:** 10.1186/s40309-022-00197-4

**Published:** 2022-05-05

**Authors:** Michel Héry, Marc Malenfer, François de Jouvenel, Laurie Grzesiak

**Affiliations:** 1 Institut national de recherche et de sécurité, rue du Morvan, CS 60027, 54519 Vandœuvre-lès-Nancy, Cedex France; 2 Institut national de recherche et de sécurité, 65 boulevard Richard-Lenoir, 75011 Paris, France; 3 Futuribles, 47 rue de Babylone, 75007 Paris, France

**Keywords:** Work organization, Covid-19, Occupational risks, Working conditions

## Abstract

The health crisis linked to Covid-19 has had the effect of strongly increasing the activity of certain trades or, on the contrary, decreasing it to the point of stopping. It has also disrupted the organization of work in companies: remote work, physical distancing, etc. The question that now arises is to know the sustainability of these developments, their influence on working conditions, and the associated occupational risks. To do this, a prospective study was conducted involving the reference body for occupational risk prevention in France (INRS), a prospective think tank (Futuribles), representatives of a dozen French companies, and a number of experts on these issues.

## Introduction

For an institute like INRS (French National Research and Safety Institute for the Prevention of Occupational Accidents and Diseases), it is important to have a vision of possible futures. This is first to help the members of the board of directors in their thinking by providing them with more systematic knowledge about possible developments in the subjects that the institute will have to deal with in the years to come. This information can help them in the choice of directions to take. The second objective is more immediate. The mutual social and technical learning processes involved in foresight work can help political decision-makers, as well as INRS researchers, to define a common vision of public policies [1, 2].

Foresight is a capacity that meets these needs quite well. The techniques used are numerous and varied [3], but the objective remains the same: to develop the ability to project oneself into the future in a rational way, taking into account the evolution of the main parameters that have a significant influence on the subject studied. This is summarized very well by Martin [4]: “Foresight is the process involved in systematically attempting to look into the long-term future of science, technology, the economy and society with the aim of identifying the areas of strategic research and the emerging generic technologies likely to yield the greatest economic and social benefits.” It is important to keep in mind that foresight is not forecasting but, first and foremost, a way of influencing the future by preparing for major eventualities [5].

INRS has been conducting foresight studies since 2013, on subjects as varied as physical assistance robots [6], the influence of ICTs on working conditions [7], and the circular economy [8]. Given its missions, all these studies focus on occupational risk prevention. INRS therefore had the expertise to consider possible adjustments to its policy and activity programs in the context of the Covid-19 pandemic. Indeed, the Covid-19 pandemic has greatly disrupted the daily lives of almost all of humanity. Work conditions have also been strongly impacted: remote work, maintaining physical distance, disinfection of workstation surfaces, etc. Companies have also been confronted with supply disruptions and have had to adopt activity management systems requiring continuous adaptations, sometimes on a daily basis [9].

Foresight studies integrating the Covid-19 health crisis are still rare. Some are devoted to questions of the sustainability of the post-pandemic society [10, 11], others to public health issues [12], or to changes in human resource management policies in the field of work [13]. We did not find any specific foresight study on the changes in work following the Covid-19 pandemic. However, outside the field of foresight, articles are beginning to be published on this subject. They often concern remote work [14, 15] but may also offer a broader view, for example, on changes in the organization of production [16, 17].

In France, a think tank and strategic foresight study center, the Futuribles International association, produced a set of contrasting scenarios for its members as early as April 2020, intended to provide information on possible developments in the global health situation through the health crisis and its management and the economic and social situation in France within 18 months [18]. This association then proposed to INRS to conduct a specific foresight study focusing on the question of work organization after Covid-19 (to 2025). The aim of the study was to provide companies and public bodies with elements that would enable them to orient themselves in the medium term to the changes in work organization brought about by the health crisis. This work was carried out between June and September 2020. It was followed by a second study at the initiative of INRS; the results of the first study were used to reflect on possible changes in working conditions and the associated occupational risks. This second study, focusing on occupational health and safety, was carried out between October 2020 and March 2021.

The results of the latter two studies, directly connected with each other, are presented in this article.

More generally, this series of two studies had two objectives. The first and most obvious one was to provide elements for reflection to both companies and specialists in occupational risk prevention regarding the evolution of work organization by 2025. Given the upheavals that the Covid-19 pandemic has brought about, reflection on its medium-term consequences on the world of work and occupational health seems useful. The second objective, of a methodological nature, was to test the possibility of using the method of contrasting scenarios, considered to be cumbersome and time-consuming, for short-term studies, and carried out under the conditions of remote collaborative work, as will be seen in the article.

### Working method

#### First study

The aim is to explore the range of possible futures via contrasting scenarios. The method used to build scenarios is morphological analysis (for more information about that method, see, for example, [19]). It has the particular advantage of presenting a whole range of possible situations (desirable or not) in an attractive and easily understandable way and of making it possible to highlight weak signals, technological discontinuities, and ruptures. It has sometimes been criticized for being time-consuming, requiring the participation of specialists in the field under investigation and possibly favoring the choice of black and white scenarios or of the most likely scenario (wishful thinking) [20]. These issues will be considered in the “Discussion” section.

A working group composed of two experts from Futuribles International, two experts from INRS, and thirteen experts representing eight French companies was set up. The participating companies had in common that most of them were of significant size (more than two-thousand employees) and operated in sectors as diverse as industry, services, transport, and local authorities. Most of the company experts were familiar with foresight and its techniques, although only a few had already participated in foresight work. A wide range of skills were represented in this group (production, human resources, research and development, futurists). This group met four times by videoconference between June and September 2020.

The first task of the working group was to obtain as accurate a picture as possible of the situation, to understand the context, and to identify the main drivers of change that are or could be influencing the issue under study. The project group therefore identified six variables for which conflicting development hypotheses were put forward. The combination of these hypotheses led to the creation of four scenarios describing possible futures.

Scenarios allow the results of a foresight study to be presented in a simple and illustrative way. In a rapidly changing context, it may be necessary to review and re-evaluate the scenarios that correspond to exploratory foresight (“what might happen?”). The objective of foresight is more ambitious; it is to identify the strategic issues that these questions pose to organizations in order to lead them to consider the best ways to answer them. This is why, in this study, the choice was made to identify, in addition, four key issues for companies. These themes, which are more general in nature, identify the trends of the last few years, the weak signals linked or not to the changes brought about by the pandemic, and the main challenges that companies will have to face in the years to come. The variables, scenarios, and themes for reflection are listed in the “Results” section, with a description of the scenarios and themes for reflection.

**Fig. 1:**
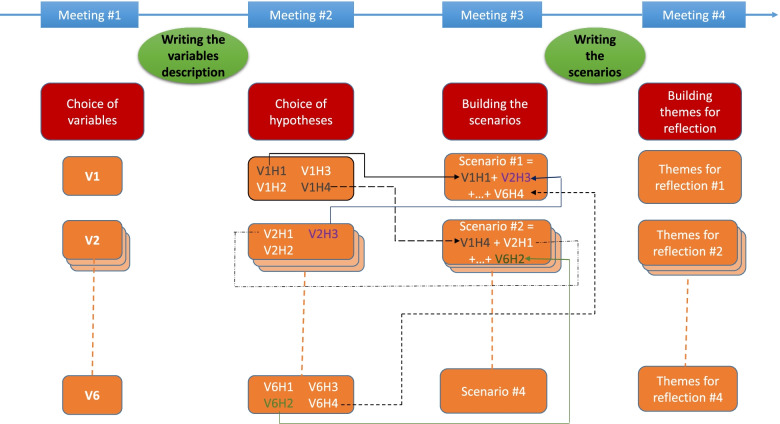
Diagram of the working method — first study

The working method used for this first study is summarized in Fig. 1.

#### Second study

This general reflection was followed by a specific study devoted to the prevention of occupational risks. The aim was to highlight possible developments linked to the crisis, in terms of risks or risk management. To this end, a specific project group was set up, composed of fourteen INRS experts (including two who participated in the first study) and three experts from official French organizations also dealing with occupational risk prevention. The current positions and initial trainings of the 17 experts involved in this second study are summarized in Table 1. All the documentations produced in the first phase of the exercise (variables, hypotheses, scenarios, themes for reflection) were provided to them. The exercise was then carried out in three steps.

**Table 1:** Field of expertise of the experts involved in the second study (determining the evolution of occupational and safety risks)

Current position	Initial training
INRS — Director of studies and research	PhD in physics
INRS — Deputy director of studies and research	MD
Enterprise A — Deputy director of a regional social security fund	Engineering
INRS — Head of medical department	MD — Occupational physician
INRS — Researcher	PhD in epidemiology and public health
INRS — Director of Paris Center	Lawyer
INRS — Foresight mission	Historian — Risk manager
INRS — Physical and psychosocial risk expert	MD
INRS — Head of technological and consulting department	PhD in chemical engineering
INRS — Health and safety consultant	Health and safety specialized graduate
INRS — Advisor at the prevention applications executive division	Engineering in mechanical development
INRS — Prevention director	General engineering
INRS — Researcher	PhD in ergonomics
Enterprise B— Deputy director of occupational risks in a social security fund	Engineering in fluid mechanics
Enterprise C — Head of studies and monitoring department	Prospectivist
INRS — Foresight mission	Engineering
INRS — External relations officer	Political sciences

The first step consisted in asking each expert to choose a subject such as an occupation, a branch of activity, an occupational risk, or an actor responsible for managing occupational risk prevention and to imagine the evolutions (relating to occupational risks or their prevention) of this subject over the next 5 years according to the four scenarios produced in the first study. The choice of topic was free. Each expert then presented his or her topic to five or six colleagues via the videoconferences and incorporated their comments. After these discussions, five topics were deemed sufficiently mature to be the subject of a written synthesis.

In the second step, each of the other four topics from the first step was discussed in more detail by five or six members of the group in four videoconferences, each devoted to one topic. At the end of this presentation, a written synthesis was made for each of these four topics.

**Fig. 2:**
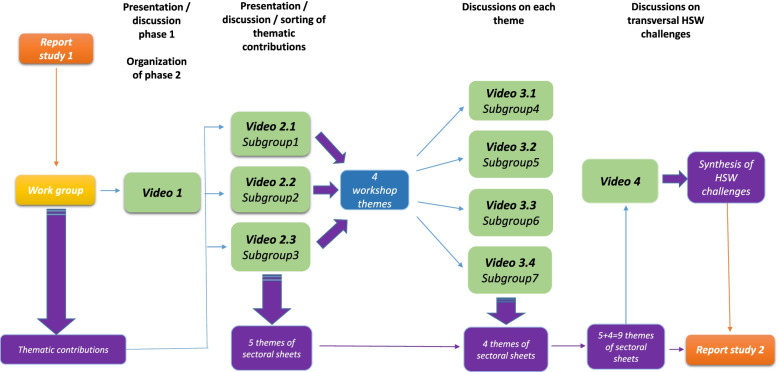
Diagram of the working method — second study

In the third step, the two INRS members who participated in the whole exercise compiled the nine syntheses and deduced six main issues in the organization and execution of work that were likely to have a strong influence on working conditions. These conclusions were validated by all seventeen experts mobilized in this second study, meeting by videoconference. The nine topics are listed in the “Results” section, where the six main issues are also summarized. The working method used for this first study is summarized in Fig. 2.

## Results

### First study — building contrasting scenarios and identifying themes for reflection

For the first study, to produce contrasting scenarios, six variables corresponding to major uncertainties for the years to come in the area of work were defined during the first meeting:

1. What role will remote work play? In what way?
2. How will work collectives and their leadership evolve?
3. How will the rules governing work relationships evolve?
4. What form will automation take in industry and services (robotization, automata, algorithmic work management, etc.)?
5. Will the criterion of social utility have an impact on the development of remuneration, recognition, and the valorization of jobs?
6. What developments in worker training can be envisaged?

The hypotheses for the evolution of these variables, the combination of which leads to the creation of four scenarios, are summarized in Table 2. The four scenarios are as follows:

- Scenario no. 1: every worker as an independent service provider in short-term organizations

**Table 2:**
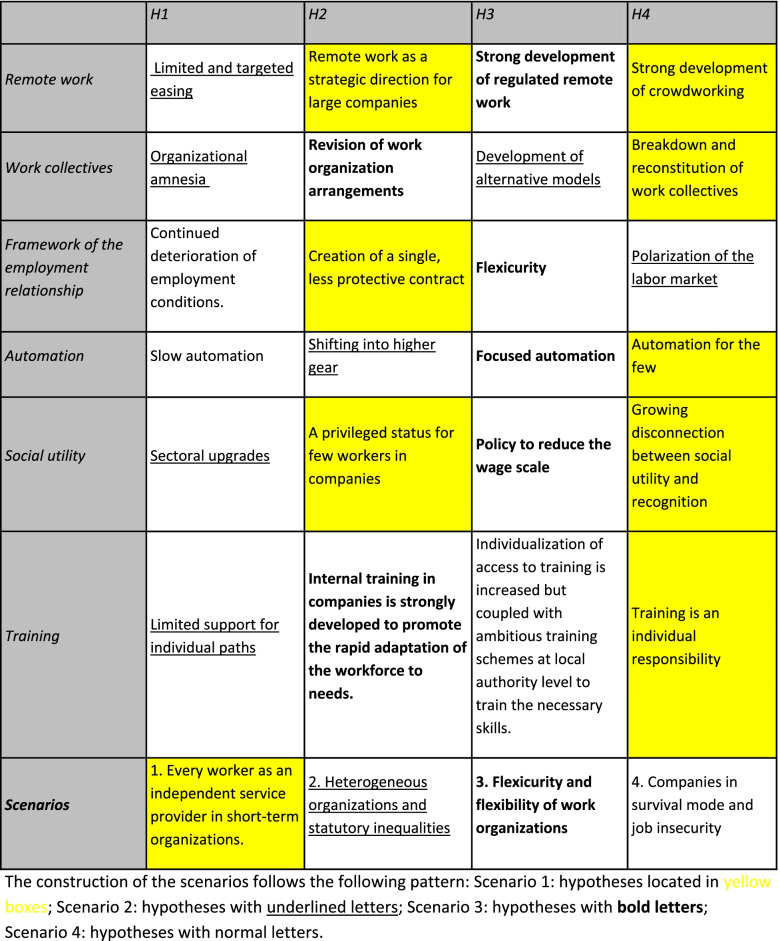
Construction of scenarios by combining hypotheses

The crisis is marked by the destruction of many jobs. After the crisis, these jobs are replaced either by automation or by hiring under precarious contracts, encouraged by regulatory changes. The aim of companies and public bodies is to become increasingly agile and able to adapt their workforce quickly. This is taking place in a context where large companies are adopting a project-based approach to which their subcontractors must adapt. These large companies are drastically reducing their own workforce and retaining workers considered essential.

- Scenario no. 2: heterogeneous organizations and statutory inequalities

After a health crisis lasting several semesters, the choices of the companies are very contrasted: some of them return to the previous model; others continue and deepen the changes initiated during the confinements. A number of workers refuse this choice and engage in initiatives aimed at building alternative models: cooperatives, freedom-form companies, etc. Inequalities and tensions increase in the social body.

- Scenario no. 3: flexicurity and flexibility of work organizations

A broad consensus between the state, companies, workers, and consumers, all committed to ecological transition, is reflected in the implementation of a flexicurity system, inspired by the Danish model [21]; it combines high mobility between jobs with a comprehensive income safety net for the unemployed and an active labor market policy, including an intensive educational policy. The labor market is more inclusive, and certain key occupations are upgraded.

- Scenario no. 4: companies in survival mode and job insecurity

The health crisis translates into a major economic crisis, the most violent of the industrial era, accompanied by a high-conflict social crisis. Companies adopt a day-to-day policy and resort to flexible solutions: the development of subcontracting and self-employment. White-collar workers are facing global competition through the development of remote work. Intermediation platforms and undeclared work are developing. In this context of strong economic crisis, some local authorities are promoting circular economy initiatives to maintain minimum cohesion in the territories.

A reflection on these four scenarios led the project group to identify four key issues on which companies may find it worthwhile to reflect in the coming years because these issues could have a strong impact on the structure of their functioning and organization.

### Remote working

Not very widespread in France before the health crisis, it has taken off significantly since. The development of ICTs, as well as urban congestion and environmental concerns, makes it a tool that can meet the aspirations of certain employees and also public policies aimed at reducing congestion, air pollution, and housing prices. They also generate savings for companies (office property). The productivity gains that seem to result from this may well be offset by difficulties in ensuring team cohesion, integrating new recruits, and maintaining the capacity for innovation [22].

### Worker and team autonomy

Up to now, organizations in silos have been in the majority, even if more and more project-based operations are introducing a little cross-disciplinarity into corporate functioning. Numerous normative processes (quality, labels, etc.) have increased the number of formal instructions that must be followed. The health crisis has shown the willingness and ability of many workers to take charge of lean systems, particularly regarding decision-making. At the same time, more and more companies are tempted by increased outsourcing and recourse to freelancers: the experience of confinements has shown their capacity to operate at a distance in a context of lean work monitoring.

### Social and environmental responsibility policies — the issue of global value chains

The crisis has shown the difficulties of managing value chains based on massive subcontracting in countries with low labor costs. This can result in technical and economic vulnerability. Many voices have called for the relocation of certain strategic production activities, some of which could be advantageously automated. Similarly, certain professions essential to the functioning of society are the subject of debate: automation and/or better recognition. In this context, corporate social responsibility and good environmental practices (reduction of carbon emissions) are no longer just window dressing but an integral part of economic strategies.

### The evolution of workers’ skills

Although it is not enough to explain the high rates of unemployment, there have been problems for years in France in matching the needs of companies with the qualifications available. Despite the fact that with the end of the health crisis we are witnessing an acceleration in the transformation of jobs, considerable efforts will have to be made regarding training schemes. It could be more difficult for smaller companies to solve their labor problems insofar as they have fewer levers for action than larger ones (attractiveness, organization of continuing training).

#### Second study — consequences for occupational risks

Initially, each expert made one or two proposals for topics. A number of these proposals were common to two or more experts. Others dealt with related topics, often generating common occupational risks that could be grouped together, for example, those concerning logistics and trade. Finally, some dealt with the same subject but seen from different angles, for example, the subject prevention policy and its actors, considered from the point of view of occupational physicians, employees’ unions, employers, or dedicated government and social security services. The latter proposals were also grouped together. This resulted in nine topics: four thematic and five sectoral.

Thematic topics:

- Topic 1: Risk perception (through reflection on risks with deferred effects)
- Topic 2: Prevention policy and its actors
- Topic 3: The issue of monitoring workers’ health
- Topic 4: Remote work and psychosocial risks

Sectoral topics:

- Topic 5: Secondary sector: industry and public works
- Topic 6: Airport activities
- Topic 7: Logistics and trade, including last-mile delivery
- Topic 8: Personal assistance and care
- Topic 9: Nursing homes for dependent elderly people

The analysis of the summaries carried out for each of these topics has made it possible to identify five main issues that will largely determine the evolution of occupational risks in the next 5 years:

1. The use of technology, as a tool for communication and collective work and for automating production (industry and services)
2. The modes of work organization, in particular notions such as working hours, task instruction methods, and agility
3. The employment status of workers
4. The ability of workers to deal collectively with work issues, particularly through the formation of sustainable work collectives
5. Controls of work interfaces multiplied by the fragmentation of tasks

A sixth issue is also likely to have a strong influence on the evolution of occupational risks, but it will not be discussed in this article since it is very specific to the French context: the national prevention policy and its actors.

### The use of technology, as a tool for communication and collective work and for automating production (industry and services)

The health crisis has greatly accelerated the use of certain technologies, such as videoconferencing, certain modalities such as medical teleconsultations, and certain sectors of activity such as e-commerce. Many workers have found themselves confronted with new ways of performing their jobs in conditions for which they were not (or were insufficiently) prepared. This has led to exposure to occupational risks that could have been avoided if the transition had been made gradually (over several months or years). One example is the various disorders linked to psychosocial risks (PSR) for some workers suddenly called to do telework. In the logistics sector, the sudden increase in orders combined with the sudden break in certain supply chains has led to exposure to risks that can result in falls or postural disorders (musculoskeletal disorders) due to the excessive pace of work, particularly in activities that are more or less robotized.

The consequences for health and safety at work will depend very much on the trade-offs that are made, but experience shows that the temptation to increase productivity at the expense of working conditions is often strong [23].

The question of the fragility of certain supply chains, in particular for certain goods considered strategic (medicines, computer components, primary metallurgy, etc.), could also be considered in the years to come. The reshoring of these production activities will inevitably be accompanied by automation, which will be all the more necessary as these activities will result in occupational exposure (e.g., exposure to metals and toxic compounds) to compounds whose presence had contributed to the decision to relocate. The developed countries might have lost the habit and competence of managing these risks. In particular, exposure during maintenance or cleaning operations, which are difficult to organize in satisfactory safety conditions, comes to mind.

### The modes of work organization, in particular notions such as working hours, task instruction methods, and agility

Remote work has helped to accentuate some of the developments that have been taking place for several years. In particular, working hours have become much more flexible over the last few decades, especially in commercial activities; the aim has been to extend opening hours (including on traditional non-working days) to facilitate consumption. During the health crisis, many teleworkers were led to adapt their working hours more or less spontaneously to the needs of the moment, resulting in a strong increase in productivity [22]. Even more revealing has been the difficulty of separating personal and professional life. It will be interesting to see whether the postcrisis period will see a return to the situation that existed before or whether new regulations or new social agreements between employers and workers will ratify the new situation and under what conditions. Here too, the transformative nature of the crisis will have to be assessed.

For several decades now, we have also seen the rise of two demands that are sometimes difficult to reconcile. On the one hand, there has been an increase in work instructions linked to standardization and quality assurance policies which have flourished in the context of more subcontracting and legal security. On the other hand, there is an increasingly strong demand for agility expressed by companies towards their workers; the latter must therefore adapt to change and promote it rapidly, develop multidisciplinarity and collaboration, and focus on creating value while ensuring the sustainability of activities. Periods of containment have shown the difficulty of achieving a number of these sometimes conflicting objectives. The likely development of remote work will certainly require adjustments to these rules. Otherwise, difficulties could arise in a context of impeded relational quality, reduced room for maneuver, inability to have objectively justified requests taken into account because of the difficulty of dialogue, and conflicts of values.

Certain deleterious situations can give rise to self-exploitation or the masking of emotions that is difficult to sustain over time. They can also lead to a generational divide; the conditions of remote work are more often unfavorable to young workers, both on the material level (inadequate housing) and on the professional level (isolation, lack of network and mentoring, etc.). Nor should we overlook the difficulties in adapting to new technologies that older categories may encounter. Similarly, gendered approaches may be relevant, for example, in relation to the education of children or the distribution of tasks. All of this can contribute to a feeling of insecurity and a mental burden detrimental to working conditions. Psychosocial problems may result.

### The employment status of workers

After a sharp decline after the Second World War, the status of self-employed worker has been revived with the development of the digital labor platform economy, in particular on-demand platforms such as Uber (Eats), Taxify, and Deliveroo. The health crisis has made clear the fragility of this status, particularly in terms of social protection, including occupational risks. This is illustrated by the case of bicycle delivery drivers, who are supposed to organize their own occupational risk prevention without having the means to do so; their activity is dictated by an algorithm that takes no account of the realities of the moment (weather, accessibility of the road network by two-wheelers, traffic conditions, etc.), which is why many accidents occur [24]. The high time pressure that these workers are under, in a context where a bad rating by the client can deprive them of their job, is also a factor of PSR.

In the care sector, where the use of temporary or even freelance workers is increasingly common, their lack of knowledge of the environment in which they are working on an ad hoc basis also results in the increased proceduralization of work for all staff (in conjunction with the development of lean management). This can result in quality hampered (the fact that the worker is unable to do his or her job properly due to lack of time or resources), a lack of recognition, and a reduction of margins of maneuver, all of which are potentially harmful for the quality of the service (including its relational aspects with patients) and for the worker (PSR).

The risk of hiring freelancers in certain activities involving assembly line work has also been identified, when it has the effect of increasing the pace of work for all those involved in the chain concerned.

The development of telework could change the legal relationships governing professional activity. An employer who meets an employee only occasionally because the latter’s work can be carried out mainly remotely may consider substituting a commercial relationship for the employer/employee relationship. The performances of ICTs now allow these contracts to be concluded across borders in countries with low wage costs, with a downward effect on wages. We are also seeing the proliferation of collaborative work platforms in which the expert, with the status of a freelancer, can intervene only for very specific missions [25]. The risk is that they will be forced into self-exploitation and will have to work in a context where they will sometimes only have a partial view of the cases they are dealing with, with all the possible consequences in terms of mental health (loss of meaning of the work) and physical health (exhaustion).

However, the self-employed status in itself may appeal to workers who are concerned about a chosen work/life balance and who, either because their high skills are in themselves protective or because they envisage such status for a limited period of their career, are not handicapped in their integration in the world of work. This situation may also be protective in terms of occupational health.

Cooperative experiments could perhaps take off in the coming years. The question of working conditions often occupies a significant place in the vision that these cooperative workers have of their work. This is obviously favorable to the prevention of occupational risks.

### The ability of workers to deal collectively with work issues, particularly through the formation of sustainable work collectives

For several years now, some employers have wanted to establish a direct and individual relationship between themselves and their employees. The aim is to bypass the intermediation provided by the trade unions but also to individualize professional objectives. These measures can have the effect of weakening work collectives, which associate workers through the sharing of job rules and work quality criteria. These collectives are built through the recognition of skills, trust, and exchanges on values [26]. They have a protective effect on workers’ health, in that they encourage consideration of real work (all the actions carried out and strategies deployed by the employee to carry out his or her activity) rather than prescribed work.

It is to be hoped, however, that the experience of the health crisis will give some impetus to “virtual” work collectives. For example, discussion forums have been set up by certain companies or spontaneously by the workers themselves on certain social networks. In the context of increasingly fragmented work, they offer a minimal alternative.

There are other parameters in the organization of work that can have a disruptive effect on these work collectives. We have already mentioned the diversity of employment contracts, especially the most precarious ones, and the use of self-employed workers. The subcontracting of activities such as maintenance, supplies and product packaging, and cleaning is another important factor. In some cases, solidarity and a common understanding of work appear over time, when external workers remain for a significant period of time in the subcontracting company, but this phenomenon is not automatic. Similarly, automation, also mentioned above, can also cut workers off from contact with their fellow workers when the relationship with the machine becomes exclusive; this is a situation in which it is often difficult to give meaning to one’s work, with possible consequences in terms of occupational risks. All these situations are likely to result in an increase in occupational risks: PSRs, of course, but also all other potential risks (chemical, physical, biological, mechanical accidents, falls, etc.).

The issue of new recruits should also be mentioned. Their integration in the company was difficult because of the health crisis. As we have seen, the transmission of knowledge is largely done informally through work collectives, regardless of the quality of the procedures used by the company. The next few years will be decisive in ensuring that the shortcomings suffered during the crisis can be eliminated.

### The control of work interfaces multiplied by the fragmentation of tasks

The previous four issues highlighted a number of parameters that strongly influence working conditions and the resulting occupational risks. These include the following:

- The diversity of employment statuses (permanent staff or fixed-term contract workers or self-employed workers, main company or subcontractor, etc.) which results in a weakening of work collectives
- Quality assurance policies and the growing trend towards instructions and standardization, while more and more companies are demanding agility.
- Automation, which sometimes places the machine at the center of the production process, with workers having to adapt to the work pace and processes that do not take sufficient account of the capacities and limits of human work

To the above can be added the gradual shift of proximity managers from production tasks to reporting tasks linked to the increase in instructions and administrative tasks.

All these elements contribute to making it even more essential to manage the interfaces between the various actors of production, an essential factor in the prevention of occupational risks [27]; it makes it possible to avoid accidents, chronic or acute exposure to pollutants, difficult postures, etc. This control implies that reflection is carried out on every activity and its phasing, and that it goes beyond the prescribed aspects to also consider real work, formal and informal communication, adaptation, and appropriation of the work instructions by the group. If the post-Covid period was to see the perpetuation of a certain number of measures taken during the crisis, such as easier recourse to temporary workers or an acceleration in the use of new technologies or automation, the risk would be that if these changes are too rapid, the real importance of this issue of interface management might be ignored. Gray areas would then appear, i.e., places of interaction where the risks linked to co-activity are significant but where the responsibilities of each party are not clearly defined. This would hinder the implementation of effective prevention (insufficient feedback on incidents, failure to record occupational exposure, lack of transparency of actions, etc.).

#### Assessment of the relevance of a remote operation compared to usual face-to-face practices

This remote prospective study was imposed by the health context. It showed very good involvement from the actors (presence at meetings, active participation in the drafting of the different documents). However, as with the face-to-face exercises, it is not possible to draw conclusions about the relevance of the different elements produced (scenarios and issues) even if, at first sight, the output products seem to be of comparable quality. Only a follow-up over time with bibliographic benchmarking will allow assessing the results of such a format.

## Discussion

### Adapting the working method of the two studies to the specific conditions of remote work

This was INRS’s first experience of conducting foresight exercises in remote work conditions. No major difficulties arose, although in the literature the method is described as requiring a considerable investment of time and the use of specialists for the issues dealt with [20]. Several particularities may explain this fluidity in the conduct of these two related studies.

For the first study:

- All the participants already had experience of what the objectives of foresight are; several of them had collaborated previously on such studies.
- Most of the variables were written by the two members of Futuribles and the two members of INRS, with technical support from experts in the group; these four people had several years’ experience in writing this type of document.
- Most of these topics had already been addressed in previous studies devoted by INRS to changes in work and its organization, in connection with the growing use of new technologies. Much of the necessary raw material was therefore available [2, 6, 8, 28] and could be adapted.

For the second study:

- Half of the experts invited had already engaged in this type of work, which aims to deduce possible consequences in terms of occupational risks and their prevention from the scenarios drafted in a foresight study; a quarter of them had already participated in foresight studies and developed variables, hypotheses, and scenarios themselves.
- The purpose of foresight work at the INRS is to promote multidisciplinarity, and this approach is strongly encouraged by the institute’s management.

It is probable that the exchanges would have been richer if all the debates had been conducted in person, but the rigor and investment shown by all the participants (familiarization with the documents produced during the intersessions before the new work session for the first study, initial reflection prior to the start of the second study leading to the drafting of the sheets, etc.) made it possible to optimize the time spent on online discussions.

Nevertheless, it is probably preferable to limit these remote exercises to subjects with which the participants are already familiar. It seems rather difficult to organize fruitful discussions between experts from different disciplines at a distance; understanding is achieved not only through words but also through attitudes, facial expressions, etc. The smaller discussions that take place during breaks also play their part in the creation and maturation of a common line of thinking.

### More and more for the customer experience?

The results of the two studies did not show anything really new compared to previous work on this subject [6, 28, 29], but they did show a very strong acceleration of previously identified transformations in certain areas. In other words, changes that had been identified as likely to occur within 5 to 10 years are now considered likely to occur very soon or have even already occurred during the health crisis, particularly during periods of lockdown. The fact that the experts involved in the first study did not imagine any new radical transformations is perhaps a methodological bias that may be due to two factors:

- Remote meetings are less disruptive than face-to-face meetings, and imagination is curbed.
- The fact that some of the material used in the first study was taken from previous work may have oriented the work of this study in a similar direction; however, this reuse concerned only some of the variables, and the reuse of each variable was only partial; moreover, the hypotheses and their combinations were the result of the work of the group of experts set up specifically for the first study.

Whatever the case, the horizon chosen (5 years) probably did not favor the choice of radical breaks in the modes and methods of production in the minds of the experts.

Examples of this acceleration of changes have already been identified: remote working, of course, but also automation in many economic sectors, both in services and in industry. For example, Microsoft boasts that it saw 2 years’ worth of digital transformation in the first 2 months of the Covid-19 crisis (https://www.microsoft.com/en-us/microsoft-365/blog/2020/04/30/2-years-digital-transformation-2-months/). Similarly, investment in robotic process automation (RPA) is expected to increase by almost 20% in 2021 compared to 2020 (https://www.gartner.com/en/newsroom/press-releases/2020-09-21-gartner-says-worldwide-robotic-process-automation-software-revenue-to-reach-nearly-2-billion-in-2021). This automation, through the use of certain artificial intelligence (AI) techniques such as optical character recognition (OCR), relieves workers of repetitive and boring tasks and could also contribute to the enrichment of certain activities [30].

This acceleration also means that a certain number of organizational changes will become irreversible, for example, we cannot imagine significantly reversing changes that were already in the making and which implementation was accelerated by periods of confinement [31]. This is a real concern in terms of occupational risk prevention. Indeed, in times of crisis, negotiations of employers with workers and their representatives have often been limited, and it is likely that all the consequences in the evolution of work organization and their impacts on health and safety have not been completely assessed. This comes in a context where, since the first developments of the gig economy in the early 2000s, the customer experience has been at the center of every concern, often at the expense of working conditions [32]. We could therefore see a sharp increase in occupational accidents and illnesses in the coming years, before awareness dawns of the mistakes made.

The situation will certainly evolve differently in different economic sectors, and national parameters (regulation, quality of the social safety net, etc.) will also play a role, but there are elements that give cause for vigilance. One example is fast fashion, which has accelerated its strong development since the beginning of the pandemic. An independent audit, the conclusions of which were accepted by the company, showed that the organization of Boohoo’s production (entrusted to subcontractors) did not respect the minimum health and safety conditions in which workers can carry out their work without risk [33]. The relocation of production to the UK in order to be able to deliver more quickly to customers was accompanied by unacceptable forms of employment (pay below the minimum wage, unhealthy workplaces that do not allow workers physical distancing measures, etc.). The conditions of this relocation will have to be closely monitored at a time when the relocation to developed countries of certain production activities considered essential is a hypothesis for the years to come.

### Employment contracts and social protection

Studies have shown the difficulty for self-employed workers to organize their own occupational risk prevention [24]. The difference in professional status was also identified in our second study as likely to complicate the management of occupational health and safety issues because it multiplies the interfaces, dilutes responsibilities, and makes it more difficult to implement common policies between the various companies and, in particular, self-employed workers.

The development of remote work, largely facilitated by new and increasingly efficient technologies, could have the effect of distending the relationship between employer and employee, to the point where some could envisage replacing the employment contract by a commercial service contract. A departure from the classic employment relationship indeed occurs if the conditions under which the service is provided can be easily defined and the worker only needs to visit the employer’s premises occasionally. Algorithms already allow a principal to “automate” the coordination of the production of their various subcontractors [25], just as they would organize the production of their own employees.

At the same time, however, a report by the Massachusetts Institute of Technology (MIT) emphasizes that the development of technology in the coming years could be hampered not by technical problems but rather by shortcomings in human resources [34]. The training of many workers in the USA, especially women and minorities, is currently insufficient for the future needs of the labor market. The report recommends tax incentives for companies that make a significant effort to train their staff. This seems to be a fairly general trend in developed countries, although differences in educational and employment policies certainly exist [35]. In any case, it is not clear that self-employment is the best way to guarantee workers the financial means to obtain training, or that companies will easily find in such circumstances the specialists they need on the labor market. Whatever the case, from the point of view of occupational health and safety specialists, it is obvious that training adapted to the work performed is a solid basis for developing an effective occupational risk prevention policy.

### Growing inequalities between workers and within sectors and companies

One of the limitations of the first study was its general and cross-industry approach, whereas the pandemic crisis is characterized by very contrasting effects depending on the sector of activity, the profile of the companies, and their location. The qualification of workers is also a factor that should ideally have been taken into account. These shortcomings are due to the time frame for this first study, which was very short (3months) and did not allow for in-depth sectoral studies. This can also be explained by the composition of the group, the majority of whose members came from large companies, with a profile more oriented towards a broad vision of work organization issues rather than a sectoral vision by activity. During the second exercise, devoted to occupational health and safety issues, sectoral details were proposed, but without seeking to be exhaustive. It therefore remains for the users of the materials resulting from this work to transpose and adapt the issues to their activity, their environment, etc. These two studies were carried out precisely for this purpose. These reflections will benefit from being conducted collectively, with other companies in the same sector, but also with actors in their ecosystem: suppliers, subcontractors, local authorities, etc. To be truly resilient, reflection on work organizations will have to integrate the issues of interdependence and interfaces between players.

To illustrate the great heterogeneity of the impacts of this crisis, we can take the example of the catering sector, which can be considered to have been particularly affected by the health measures. However, the effects are very unequal between fast food restaurants, which were able to continue to operate with takeaway sales and deliveries, and collective catering, which was often shut down in companies. Users of our studies must also consider the overall environment, taking into account the dependency of companies on each other, including for future developments in the next 5 years; foresight is a good tool for this purpose.

## Conclusion

### Relevance of remote work for the realization of a prospective exercise using the method of contrasting scenarios

This work has shown that it is possible to carry out foresight exercises using the INRS reference method (that of contrasting scenarios) in a short and remote format. This choice was certainly imposed by the circumstances of the health crisis, but these lessons are valuable. Indeed, over the years, foresight has become an important tool for INRS to initiate multidisciplinary reflections on new subjects by contextualizing them in the possible evolution of the world of work and its organizations. The possibility of doing this at reduced cost will make it possible to reiterate such preliminary studies.

The two studies described in this article benefited from the results of previous work, and it is difficult to ascertain whether the task would not have been much more complicated for the teams involved if they had to focus on a completely new subject. Exchanges are much more complicated via videoconferencing devices. There is also a risk that they will be more superficial. This work also benefited from the strong commitment of all the persons solicited; several of them indicated that the conditions of relative isolation with which they were confronted because of the health lockdowns acted to spur their resolve to carry out prospective work, which is by essence very collaborative.

The relevance of our work can be judged in the long term; as with all the other exercises carried out by INRS [36], monitoring will be implemented for the main developments recorded in the coming years relating to the main key issues identified.

### Some lessons that can be learned from this work by companies

It must be emphasized that these results are not an end in themselves; they are an invitation to companies to continue reflection by adapting these results to the context in which they are evolving. To be effective, this reflection must adopt one of the principles of foresight: the ability to build relations and interactions between the different elements of the context and also the ability to estimate the pace of changes and to link short-, medium-, and long-term evolutions. Companies must therefore be able to reflect on the different interactions they have with their ecosystem: professional branch, subcontractors, etc.

More generally, two elements seem to have met with a broad consensus during the two studies:

1. The health crisis will act as an accelerator of changes that have often been initiated in recent years: automation in both industry and services, the presence of algorithms in work activities, employment contract flexibility, and, thanks to these elements, the relative reshoring to developed countries of certain activities that were previously relocated to countries with low labor costs.
2. All these changes will also have the effect of accelerating the hybridization of the gig economy with the more traditional forms of business (brick and mortar); a number of mergers that were already underway before the health crisis have accelerated and are likely to continue in the postcrisis period. The consequences in terms of work organization and occupational risks are therefore going to be significant, since the initial models are very different.

The real novelty of these changes is their acceleration. Indeed, the growing importance of automation, the influence of the gig economy on the operation of other companies, and the changes in workers’ status had already been identified in previous studies [7, 26, 27, 34]. The results of the present study constitute an incentive to reinforce the documentary monitoring carried out on these subjects by the INRS, in particular to identify new consequences for occupational risks. The combination of monitoring and foresight is an effective tool for identifying, in advance, changes in working conditions that will result in significant changes in terms of occupational risks. On one hand, the results of monitoring (e.g., weak signals) help guide the choice of new foresight topics. On the other hand, the results of foresight provide elements for selecting topics of monitoring on working conditions that are likely to have an impact on health and safety.

These changes are also likely to be very important in terms of social protection. Until now, in the majority of European countries, this social protection (of which occupational risk prevention is part) was associated with work and financed by a contribution from companies. The development of self-employment, if it continues, could contribute to calling this model into question. In the case of occupational risk prevention, it is not only a question of financial balance but of the capacity (or inability) to sustain a system designed on the basis of an ambitious conception of health and safety at work involving the treatment of problems based on the pooling of knowledge and actions. Although this system obviously has imperfections, it has the merit of identifying responsibilities and involving them in the implementation of solutions that are valid for all stakeholders, regardless of their status. The generalization of the transformation of employment contracts into economic service contracts would radically change the situation.
